# Unstimulated inflammatory activity is associated with treatment response to cognitive-behavioral therapy for urologic chronic pelvic pain

**DOI:** 10.3389/fpain.2025.1593807

**Published:** 2025-09-17

**Authors:** L. C. McKernan, L. J. Crofford, S. Bruehl, T. W. McGonigle, A. G. Kelly, A. M. Ryden, S. L. Sutherland, D. J. Clauw, D. A. Williams, R. R. Dmochowski, A. D. Schrepf

**Affiliations:** ^1^ Department of Psychiatry & Behavioral Sciences, Vanderbilt University Medical Center, Nashville, TN, United States; ^2^ Department of Physical Medicine & Rehabilitation, Vanderbilt University Medical Center, Nashville, TN, United States; ^3^ Department of Urologic Surgery, Vanderbilt University Medical Center, Nashville, TN, United States; ^4^ Department of Medicine, Vanderbilt University Medical Center, Nashville, TN, United States; ^5^ Department of Anesthesiology, Vanderbilt University Medical Center, Nashville, TN, United States; ^6^ Department of Biostatistics, Vanderbilt University Medical Center, Nashville, TN, United States; ^7^ Department of Psychology, George Mason University, Fairfax, VA, United States; ^8^ Department of Anesthesiology, University of Michigan, Ann Arbor, MI, United States

**Keywords:** interstitial cystitis, pelvic pain, UCPPS, inflammation, Toll-like receptor, cognitive behavioral therapy, psychotherapy

## Abstract

**Introduction:**

Interstitial cystitis/bladder pain syndrome (IC/BPS) is a debilitating urologic chronic pelvic pain condition characterized by pelvic pain and urinary symptoms. Evidence suggests that in chronic pain conditions such as IC/BPS, inflammatory markers are associated with heightened symptom severity and widespread pain. Non-pharmacological treatments such as cognitive-behavioral therapy are recommended as a core component of IC/BPS treatment. There is limited and mixed evidence as to whether inflammatory markers are affected by non-pharmacological treatments or their relationship to treatment response. This exploratory study considered how inflammatory characteristics may both predict and explain treatment response in a sample of females with interstitial cystitis.

**Method:**

Participants were randomized to receive either 8-weeks of telemedicine-delivered cognitive-behavioral therapy (CBT) or an active attention control. Six cytokine/chemokines in whole blood plasma (IL-6, IL-8, IL-10, IL-1β, and TNF-α) were assessed in a subset of trial participants at baseline, post-treatment, and at five months. We assessed relationships between baseline plasma inflammatory cytokine levels and self-reported symptoms, changes in cytokines over time, and how baseline cytokine levels may relate to clinically meaningful indicators of change following CBT.

**Results:**

Cytokine/chemokine levels did not significantly change over time. Higher levels of unstimulated IL-1β were associated with significantly worse clinical pain characteristics and greater degree of CBT treatment response.

**Discussion:**

This suggests that individuals with greater degrees of inflammation may derive more benefit from the self-regulation training, pain coping strategies, and cognitive reframing offered in CBT for pain.

## 1 Introduction

Interstitial cystitis/bladder pain syndrome (IC/BPS) is a debilitating urologic chronic pelvic pain condition characterized by pain, pressure or discomfort in the pelvis, and urinary urgency, and frequency that affects up to 10 million individuals in the United States. Treatment has been hindered by issues of how IC/BPS is defined and a lack of consensus regarding its pathophysiology. Consequently, efforts to advance existing treatments and understand their mechanisms have stalled and not matched the service demand. For instance, the only medication approved for IC/BPS, pentosan polysulfate sodium (Elmiron®), was approved more than 25 years ago and has demonstrated limited efficacy and serious side effects (1). Alternative pathophysiologic frameworks are needed to advance the understanding of IC/BPS. Recent work demonstrates that dynamic measures of *ex-vivo* inflammatory activity (e.g., stimulated cytokine release) appear to be associated with nociplastic pain symptoms in IC/BPS—symptoms primarily driven by aberrant pain-processing in the central nervous system (CNS)—with a clear impact on functional and structural brain measures.^1^ However, the role of unstimulated circulating inflammatory markers remains less clear in IC/BPS, including whether they may influence treatment responses.

Research has demonstrated the role of dysregulated inflammation in IC/BPS. Investigations previously reported elevation in stimulated *ex-vivo* pro-inflammatory and anti-inflammatory cytokines in IC/BPS. These findings echo studies of irritable bowel syndrome (2) and other pain conditions (3–5) demonstrating enhanced cellular immune responses. Studies also show elevated unstimulated levels of interleukin-6 in IC/BPS [IL-6; (6, 7)]. While the relationship between *ex-vivo* inflammatory markers and nociplastic pain symptoms has been replicated in IC/BPS, it is less clear whether these markers relate to index symptoms of IC/BPS, such as genitourinary pain (6, 7).

Behavioral (psychosocial) interventions are a cornerstone of IC/BPS management per national guidelines (8). Cognitive-behavioral therapy (CBT) is a gold-standard psychosocial intervention for chronic pain (9) where individuals work with a provider to reduce pain and distress, enhance coping skills, and build self-efficacy in managing symptoms. Coping strategies can target emotional and behavioral responses to symptoms, physiological downregulation, thought patterns influencing pain, and tools to address other stressors affecting a person's quality of life (e.g., assertive communication, problem-solving techniques) (10). Preliminary evidence suggests that CBT can downregulate inflammatory cytokines in chronic pain, although this is a highly novel area of study with very limited available information. There is some evidence that CBT may reduce inflammatory cytokines in individuals with co-morbid depression and chronic conditions such as heart disease, rheumatoid arthritis, and irritable bowel syndrome (11). Drawing meaningful conclusions from existing investigations is limited by their lack of comparators, randomization, or assessment of confounding factors that may affect both systemic inflammation and treatment outcomes [e.g., obesity (11)]. More randomized controlled trials are needed to better understand how inflammation may relate to CBT outcomes over time.

This preliminary prospective study assessed the potential role of inflammation in both predicting and explaining symptom improvement following CBT tailored to IC/BPS. First, we sought to evaluate the relationship between inflammatory markers and baseline genitourinary symptoms. Second, we assessed if there is evidence inflammatory characteristics may change after CBT or are associated with patients’ subsequent global perceived response to treatment. Lastly, we explored whether any observed change in inflammation was associated with meaningful global, pelvic pain, or urinary symptom changes reported by participants post-treatment.

## 2 Method

### 2.1 Study protocol

This study is a secondary analysis of a randomized controlled trial (NCT#04275297) assessing the potential for a tailored individual CBT program to improve outcomes in IC/BPS [see (12) for full protocol]. Informed consent was obtained for all study participants. The primary study involved an unblinded, parallel group prospective design with participants assigned to receive either an 8-week CBT program tailored for IC/BPS or 8 weeks of symptom monitoring telephone calls and randomized 2:1. For this project, we analyzed a subset of female study participants who underwent additional in person assessment visits providing data on IC/BPS phenotypic characteristics including evaluation of inflammatory cytokines in whole blood samples. Participants completed study visits pre-treatment (*n* = 39), immediately post-treatment (*n* = 35), and at three months post-treatment (*n* = 30).

All study procedures were approved by the institutional review board and conducted in accordance with the Declaration of Helsinki. Data collection occurred 7/2020–6/2022. At each of the three assessment timepoints, in addition to the battery of validated self-report measures assessing symptoms, physical health, and emotional wellness [see (12) for full details], participants attended a 60–90 min in-person assessment that included providing urine and blood samples and undergoing a quantitative sensory testing protocol (not reported).

### 2.2 Inflammatory activity procedures and assay

Collection of whole blood occurred via a single venipuncture blood draw. A trained nurse collected a maximum of 12cc of blood, using two lavender K2-EDTA 4 ml vials for blood plasma collection. Following their collection, tubes were gently inverted 8 times, with lavender K2-EDTA tubes centrifuged immediately at 2,500 g for 15 min. Plasma supernatant was then transferred into pre-labeled cryotubes in 1 ml aliquots and frozen at −80 degrees.

Assays of inflammatory cytokines were conducted with Luminex multiplex xMap technology via the MagPix system (Thermo Fisher Scientific, Waltham, MA). Undiluted, thawed supernatant of blood plasma was analyzed for the following six cytokines/chemokines collected at each timepoint with Millipore systems high performance assays: macrophage inflammatory protein 1-α (range of assay: MIP-1α, 0.31–1,250 pg/ml), IL-1β (0.49–2,000 pg/ml), IL-6 (0.18–750 pg/ml), IL-8 (0.31–1,250 pg/ml), IL-10 (1.46–6,000 pg/ml), and tumor necrosis factor-α (TNF-α; 0.43–1,750 pg/ml). These analytes represent pro- and anti-inflammatory aspects of the inflammatory response and are all regulated by the NFκB axis (13–15). See Table 1 for intra and inter-assay coefficients of variation.

**Table 1:** Intra and inter-assay ^a^CVs for each analyte.

Cytokine/chemokine	Variation	
	Intra-assay CV %	Inter-assay CV %
I-10	5.70	5.72
IL1-ϐ	2.48	3.56
IL-6	7.27	6.90
IL-8	6.40	5.49
MIP1α	3.31	4.06
TNF-α	3.39	5.59

^a^ CV, coefficient of variation.

All samples were run in duplicate with average values used for analysis. All values were above the limit of detection and were included in the analysis.

### 2.3 Patient-reported outcome measures

Participants completed the following selection of patient-reported outcomes. We assessed relationships between these measures and inflammatory markers at pre-intervention baseline and over time, in response to treatment:

#### 2.3.1 Demographics and clinical information

Participants completed a brief self-report questionnaire indicating their demographic characteristics including age, sex assigned at birth race, relationship status, employment status, current opioid use, smoking status, and age of diagnosis and symptom onset. Height and weight characteristics were recorded by nursing staff at the beginning of the in person visit. For full sample characteristics please see McKernan et al. (12).

#### 2.3.2 Global impression of change [PGIC (16)]

Participants reported their global impressions of change immediately post-treatment and at 3-month follow-up. This measure includes a single item rating how much participants believe their symptoms have improved since initiating treatment on a 7-point Likert scale (0 = “no change or condition has gotten worse” to 7 = “a great deal better and considerable improvement”). This scale is dichotomized into treatment responders at a threshold of ≥ 5 (“moderately better”).

#### 2.3.3 Genitourinary symptoms [GUPI (17)]

The genitourinary pain index assessed participants pelvic pain severity, urinary symptom severity, and quality of life impact due to symptoms at all timepoints. While a total score indicates overall symptom severity, recent investigations urge the assessment of pain and urinary symptoms separately due to heterogeneity of symptom presentations (18). As such, all three scales are reported in the current study, with meaningful change in urinary symptom severity (USS) and pelvic symptom severity (PSS) estimated in accordance with recent recommendations on clinically meaningful response thresholds for clinical trials [USS *Δ* 3 for females, PPS *Δ* 4 (19)].

#### 2.3.4 Pain intensity [NRS-11 (20)]

Pain intensity was measured with a subscale from the PROMIS-29-2.0 containing a single item 11-point Numeric Rating Scale (NRS). Higher scores indicate greater average pain intensity in the past week.

#### 2.3.5 Pain interference [PROMIS (21)]

Pain interference was measured via the PROMIS-29-2.0. This four-item scale assesses the degree to which pain has interfered with daily activities, work, social activities, and completion of household chores in the past week. Higher scores indicate greater interference.

### 2.4 Statistical analysis plan

All cytokine values were transformed on a natural-log scale so they would be appropriate for parametric statistical tests. To determine if levels of cytokine/chemokines were associated with symptoms at study entry, we conducted Pearson correlations between log-transformed cytokine/chemokine values and self-reported pain and genitourinary symptoms. To determine if treatment had an impact on cytokine/chemokine levels, we conducted a repeated-measures ANOVA with a time X treatment group interaction effect for each cytokine./chemokine. Multiple corrections were applied to the resulting tests. To determine if any cytokine/chemokine values were associated with the likelihood of achieving a clinically meaningful response to CBT, we compared each of the cytokine/chemokines by responder status on the PGIC (≥5, “moderately better”), urinary symptom severity (USS > *Δ*3), and genitourinary pain symptoms (PPS > *Δ*4), using one-way ANOVA. Responder thresholds followed guidelines for meaningful change in urologic symptoms for females (19).

## 3 Results

### 3.1 Participant characteristics

Participants were 39 individuals of female sex who completed the CBT intervention (*n* = 28) or control condition (*n* = 11) and provided blood for analysis of cytokines/chemokines at the baseline visit. Most of the sample identified as women and one individual as a transgender man. The average age was 46.2 years (SD = 14.7), with individuals reporting an average age of symptom onset at 27.6 years (SD = 14.0). The average BMI was 31.14 (SD = 8.24). The sample was predominantly white (88%) and married/partnered (65%). Five individuals (5/34, 14.7%) held opioid prescriptions, and no individuals smoked. Approximately half (47.6%) of the sample worked full-time, with 10 individuals (29.4%) reporting unemployment or an inability to work due to their symptoms.

Regarding pain and urologic symptoms, the average female genitourinary pain index (GUPI) score was 26.9 (SD = 8.9), consistent with the elevated symptom severity expected in a urologic pelvic pain sample. Pain NRS scores (range 0–10) were moderate intensity on average [4.8 (SD = 2.3)] and average PROMIS pain interference raw scores were 10.2 (SD = 4.4), translating to an average T-score of 57.5 (SD = 7.6). The full original study sample is previously described (12).

### 3.2 Associations between baseline cytokine/chemokine levels and clinical symptoms

There was a consistent, significant association between clinical pain characteristics (*r* value range.492-.533, all *p* < 0.05) and log-transformed values of IL-1β (Table 2). There was a marginal non-significant association between IL-1β and urinary symptoms (*r* = .364, *p* = .068). We observed no statistically significant relationships between other inflammatory markers and baseline symptoms. Sensitivity analyses using BMI, depression levels [PHQ-8 (22)], or anxiety severity [GAD-7 (23)] in a partial correlation framework did not substantially change the results (see Supplementary Table S1 for partial correlation coefficients).

**Table 2:** Associations between baseline cytokine/chemokine values and clinical symptoms of urologic pelvic pain.

Cytokine/ chemokine	Clinical symptom					
	^a^GUPI pain	GUPI ^b^QOL	Total GUPI	GUPI urinary	Pain ^c^NRS	Pain interference
^d^IL-6	0.086	0.034	0.035	−0.090	0.173	.052
IL-10	0.356	0.339	0.342	0.136	0.373	.166
IL-1β	0.494*	0.505*	0.533*	0.364	0.492*	.191
IL-8	0.173	0.151	0.151	0.029	0.281	.095
^e^MIP-1α	0.255	0.154	0.228	0.135	0.330	−.155
^f^TNFα	0.064	−0.005	0.065	0.110	0.165	−.254

* Significant association (*p* < .05). ^a^GUPI, genitourinary pain index; ^b^QOL, quality of life; ^c^NRS, numeric rating scale; ^d^IL, interleukin; ^e^MIP, macrophage inflammatory protein; ^f^TNF, tumor necrosis factor.

### 3.3 Longitudinal changes in cytokine/chemokine values

There were no significant treatment group by time interaction effects for any of the cytokines/chemokines tested (all *p* > .05 both corrected and uncorrected for multiple comparisons). See Table 3 for means, standard deviations and test statistics for interaction terms from each model.

**Table 3:** Means/^a^SDs and test statistics for treatment by time interaction for each cytokine.

Cytokine/ chemokine	Timepoint		Test statistic		
	pre	post	df	f	p
^b^IL-6	1.093 (0.705)	1.059 (0.706)	1	0.632	0.433
IL-10	1.374 (0.549)	1.426 (0.464)	1	0.088	0.769
IL-1β	0.475 (0.339)	0.525 (0.282)	1	0.654	0,425
IL-8	1.217 (0.634)	1.211 (0.629)	1	0.628	0.434
^c^MIP-1α	1.233 (0.219)	1.271 (0.196)	1	0.263	0.612
^d^TNFα	0.935 (0.210)	0.965 (0.202)	1	0.156	0.696

^a^ SD, standard deviation; ^b^IL, interleukin; ^c^MIP, macrophage inflammatory protein; ^d^TNF, tumor necrosis factor.

### 3.4 Relationships between baseline variables and treatment responder status in CBT group

**Figure 1:**
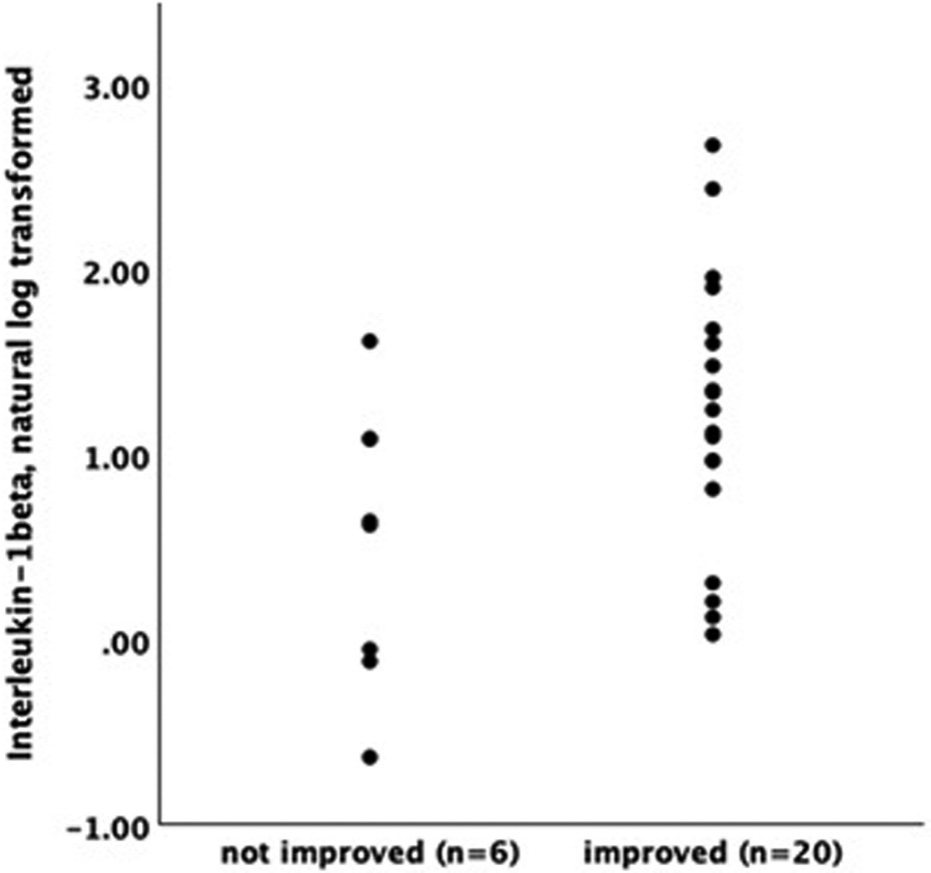
1 Baseline IL1-β by responder status.

CBT recipients who reported meaningful improvement on the PGIC (≥5) and urinary symptom severity scales (≥3) had higher levels of baseline log-transformed IL-1β (PGIC responder mean/SE = .561 +/.068, non-responder = .271 +/.124; urinary responder = .682+/.081, urinary non-responder.372 +/.088; both *p* < .05, Figure 1). This finding was not impacted by inclusion of baseline urinary severity in the model (both p from adjusted models <.05). There was no significant association between baseline levels of IL-1β and meaningful change in pelvic pain severity following CBT.

## 4 Discussion

In this set of secondary analyses, we demonstrate a relationship between symptom severity in IC/BPS and levels of the pro-inflammatory cytokine IL-1β, confirming the results of previous studies showing that higher levels of inflammatory markers measured in blood are associated with more severe urologic pain symptoms (7). Previous work has focused on immune profiles under *ex-vivo* stimulated conditions when whole blood or isolated peripheral blood mononuclear cells are challenged with lipopolysaccharide. The current work demonstrates that levels of the unstimulated pro-inflammatory cytokine IL-1β are associated with worse painful symptoms and quality of life in patients with IC/BPS. Additionally, this preliminary work shows that patients who had higher levels of IL-1β prior to initiating a course of CBT reported greater global post-treatment improvement and meaningful change in their urinary symptoms.

IL-1β is a pro-inflammatory, critical cytokine in the acute phase of inflammatory response. An optimal immune response requires the appropriate interaction between the immune system's innate and adaptive arms, as well as well-proportioned activation and regulation levels (24). Higher levels of IL-1β may reflect more global inflammatory dysregulation, and is associated with increased fatigue and symptom severity in other pain conditions (25). Elevation of IL-1 can also induce what is known as “sickness behavior,” or a behavioral response including reduced activity, low mood, and tiredness (26). This inflammatory marker may serve as a proxy for generalized worse physiological symptoms that have more potential for symptom improvement following behavioral intervention.

It is possible that CBT may have direct or indirect effects on the global dysregulation seen with higher IL-1β. CBT has previously been shown to impact the human stress response at both physiological and behavioral levels. As examples, CBT appears to result in greater cortisol habituation than waitlist controls (27), and can affect sleep outcomes, depression, and anxiety in chronic pain conditions (28, 29). Individuals with greater physiological dysregulation could derive more benefit from CBT skills that target down-regulation of the hyperactive stress response seen in chronic pain (30), such as the relaxation training or emotion regulation strategies this protocol offered. This hypothesis awaits formal testing in future work.

Of interest regarding interpretation of the current findings, one pilot study of inflammation as a predictor of CBT efficacy in chronic pain patients showed the opposite effects as those found here—higher levels of unstimulated inflammation were associated with less improvement in pain intensity following treatment. However, that study did not measure IL-1β and the sample was of mixed pain etiology that may reflect different underlying biology (31). Two other existing investigations have reported inflammation as a predictor of poor response to CBT in uncontrolled studies targeting depression (32, 33). Variable methods, different target populations, and the lack of randomization in existing investigations indicate the need for additional research.

We did not find evidence that the intervention changes levels of cytokines/chemokines over time. Although this is contrary to some preliminary investigations indicating potential anti-inflammatory effects of CBT for depression (11, 34), given the preliminary nature of this investigation, additional investigation is needed with sample sizes capable of detecting potential treatment effects. Within IC/BPS there are phenotypes (subgroups) of the condition that are more characterized by widespread vs. localized pain. Re-analyses of existing IC/BPS trials recently demonstrated that pain phenotypes respond differently to treatment (35). Immune response can also differ by sex (36). It is possible that CBT may have different effects on inflammation by pain phenotype or by sex, which requires additional investigation.

This study is a small, exploratory investigation of the concepts described in an under-studied area of chronic pain and urologic research. More specifically, a subset of the full trial sample completed in person phenotyping visits for this project, which was conducted during the pandemic. Further, randomization to treatment at a 2:1 rate limited the number of control participants for this specific analysis. Due to resource constraints, the number of cytokines/chemokines assayed was relatively small and focused on well-described inflammatory pathways; more comprehensive proteomic approaches that include additional cytokines/chemokines associated with modulating inflammation (e.g., MCP-1, IFN-g) may reveal additional insights in future, larger investigations.

The circulating inflammatory marker IL-1β is associated with worse symptoms in IC/BPS but a greater likelihood of improvement following a CBT intervention. The relationship between symptoms, inflammation, and treatment outcomes should be further explored in larger longitudinal studies.

## Data Availability

The raw data supporting the conclusions of this article will be made available by the authors, without undue reservation. Requests need to be in writing to the corresponding author, and will be honored in line with institutional policy.

## Footnotes

^1^Schrepf A, Kaplan C, Lenert M, Naliboff B, Kutch J, McKernan L, et al. Immunologic Subtypes of Women with Chronic Pelvic Pain: Impacts on Neurobiology, Pain, and Healthcare. under review (2025).
