# Supplementary material for: Unstimulated inflammatory activity is associated with treatment response to cognitive-behavioral therapy for urologic chronic pelvic pain
Source: Front Pain Res (Lausanne). 2025 Sep 17;6:1593807. doi: 10.3389/fpain.2025.1593807 (PMC12484211; doi:10.3389/fpain.2025.1593807)
Supplement: Supplementary file 2 [file Table1.docx]

| Supplemental Table 1. Partial correlation coefficients between IL-1β and symptoms at baseline, controlling for BMI, anxiety, and depression. | | | | | | |
| --- | --- | --- | --- | --- | --- | --- |
|  | GUPI pain | GUPI QOL | Total GUPI | GUPI urinary | Pain NRS | Pain interference |
| BMI |  |  |  |  |  |  |
| r | 0.508 | 0.529 | 0.562 | 0.400 | 0.519 | 0.252 |
| p | 0.010 | 0.006 | 0.003 | 0.047 | 0.008 | 0.224 |
| Anxiety |  |  |  |  |  |  |
| r | 0.412 | 0.450 | 0.460 | 0.308 | 0.413 | 0.095 |
| p | 0.041 | 0.024 | 0.021 | 0.134 | 0.040 | 0.652 |
| Depression |  |  |  |  |  |  |
| r | 0.444 | 0.459 | 0.500 | 0.399 | 0.453 | 0.150 |
| p | 0.026 | 0.021 | 0.011 | 0.048 | 0.023 | 0.473 |
