# Supplementary material for: Unstimulated inflammatory activity is associated with treatment response to cognitive-behavioral therapy for urologic chronic pelvic pain
Source: Front Pain Res (Lausanne). 2025 Sep 17;6:1593807. doi: 10.3389/fpain.2025.1593807 (PMC12484211; doi:10.3389/fpain.2025.1593807)
Supplement: Supplementary file 1 [file Datasheet1.pdf]

VANDERBILT UNIVERSITY MEDICAL CENTER

Osher Center for Integrative Medicine

Tony L. Yaksh, PhD
Editor-in-Chief, Frontiers in Pain Research
Department of Anesthesiology
University of California, San Diego
San Diego, CA

5 August 2025

Dear Dr. Yaksh and Dr. Domenichiello:

My colleagues and I are pleased to submit our revised manuscript, "Unstimulated inflammatory activity is associated with treatment response to cognitive-behavioral therapy for urologic chronic pelvic pain" for reconsideration to *Frontiers in Pain Research* as a brief research report. This data reflects an exploratory analysis examining potential predictors of response to non-pharmacological intervention in a debilitating and grossly understudied pain condition, which we believe fits well with both the journal's scope and specific article type as a brief report. We appreciate the opportunity to respond to Reviewer 2's comments and have provided a revised manuscript along with an expanded Table (3) for the Reviewer's consideration.

Thank you again for your consideration of this work and we look forward to hearing from you.

Sincerely,

Lindsey C. McKernan, Ph.D. MPH
Associate Professor of Psychiatry & Behavioral Sciences, Physical Medicine & Rehabilitation
Vanderbilt University School of Medicine
Nashville, TN

3401 West End Avenue
Suite 380
Nashville, TN 37203

tel 615.343.1554
fax 615.936.6144
www.VanderbiltHelth.com/Osher
